# Home‐Based Testing as an Approach to Estimate Influenza Vaccine Effectiveness in South Africa, 2021–2022—A Pilot Study

**DOI:** 10.1111/irv.70034

**Published:** 2024-12-08

**Authors:** Jocelyn Moyes, Mvuyo Makhazi, Sibongile Walaza, Phiwokuhle Ntombela, Fahima Moosa, Anne von Gottberg, Nicole Wolter, Mignon du Plessis, Gillian Hunt, Cherie Cawood, Erica Dueger, Cheryl Cohen

**Affiliations:** ^1^ Centre for Respiratory Diseases and Meningitis National Institute for Communicable Diseases (NICD) of the National Health Laboratory Service Johannesburg South Africa; ^2^ School of Public Health, Faculty of Health Sciences University of the Witwatersrand Johannesburg South Africa; ^3^ School of Pathology, Faculty of Health Sciences University of the Witwatersrand Johannesburg South Africa; ^4^ BARC Laboratories Johannesburg South Africa; ^5^ Epicentre Health Research Hillcrest South Africa; ^6^ Sanofi S.A. Paris France

**Keywords:** influenza, influenza vaccine effectiveness, home‐based self swabbing

## Abstract

**Background:**

Surveillance programmes for influenza and other respiratory pathogens are important to generate vaccine effectiveness (VE) estimates and to inform vaccine composition. We aimed to explore the feasibility and acceptability of home‐based testing.

**Methods:**

In three out of nine provinces in South Africa, we established a self‐referral system for individuals aged ≥ 18 years with respiratory symptoms of ≤ 10 days duration. Following consent, swab collection material was delivered to participants who also completed a questionnaire including self‐reported vaccination status. Swabs were tested by PCR for influenza, respiratory syncytial virus (RSV) and SARS‐CoV‐2. A test‐negative methodology was used to estimate influenza VE.

**Results:**

Of 1456 samples collected between 19 November 2021 and 3 September 2022, 73 (5%) tested positive for influenza, 38 (3%) tested positive for RSV and 394 (27%) for SARS‐CoV‐2. We subtyped 55% (40/73) of the influenza positive specimens; 16/40 (40%) were influenza A(H1N1)pdm09; 10/40 (25%)A(H3N2)) and all 14/40(35%) influenza B were B/Victoria. Only 20% (279/1451) of participants reported influenza‐like illness case definition symptoms of fever and cough. Influenza vaccine coverage was 11% (157/1454). The overall influenza VE was 26% (95% confidence interval: −73%, 69%). Of the completed acceptability questionnaires, 123/127 (97%) participants would make use of the service again; 90% (1306) were recruited via the COVID‐19 testing centre (call in, social media, webpage), and 7% (99/1306) through *CoughWatchSA*.

**Conclusions:**

Home‐based swabbing was feasible and acceptable. We were able to calculate an influenza VE, although a larger sample size and verification of vaccine status may improve the VE estimates in the future.

## 1 Background

Surveillance for respiratory pathogens is important to describe the circulation of viruses, inform vaccine composition, and assess vaccine effectiveness. During the height of the COVID‐19 pandemic, in 2020 and 2021, facility‐based sentinel surveillance programmes were not as effective due to fewer people accessing care in facilities [1]. Even prior to the COVID‐19 pandemic, up to 75% of episodes of mild respiratory illness were not medically attended [2]. Restricting surveillance programmes to people who seek care for illness may not be fully representative of the spectrum of people infected with influenza. The proportion of influenza illness that is medically attended may also change substantially when there are changes in health‐seeking behaviour, for example, in a pandemic. Including nonmedically attended influenza in estimates of influenza vaccine effectiveness may help to provide evidence on the full benefit of influenza vaccination.

In South Africa, annual seasonal influenza epidemics occur during the winter and spring months (May to October) and result in an estimated 19 million symptomatic infections, 128,000 severe cases, and 11,000 deaths on average every year [2]. Although a vaccine is available for influenza, the formulation is reviewed and updated annually. There is a need for ongoing monitoring of circulating influenza viruses to describe the annual circulating viruses, which will inform decision‐making related to what strains to include in vaccine formulations. Annual vaccine effectiveness estimate will assist with review of the new vaccine formulation performance. This requires robust surveillance systems that are able to access vaccinated populations. Ideally, these surveillance systems are simple, flexible, sensitive, and cost effective.

The South Africa National Department of Health purchases approximately 800,000 influenza vaccine doses annually that are distributed across the country to primary healthcare clinics and administered to at‐risk groups as defined in the influenza guidelines: people ≥ 65 years of age, people living with HIV and tuberculosis, people with chronic medical conditions, and healthcare workers [3]. The vaccine coverage in the public health sector is very low due to this limited supply of vaccines. In South Africa, influenza vaccine coverage is highest in the insured population (approx. 16% of the total population) [4], who are able to access vaccination through their health insurance or purchase the vaccine.

From the COVID‐19 pandemic, we learnt that surveillance systems can be adapted quickly. An example of this is the shift to new approaches for respiratory virus testing, including nasal swabbing and self‐swabbing (as opposed to the traditional nasopharyngeal swab collected by a healthcare worker). This, coupled with changes in health‐seeking behaviour observed during the pandemic, led to the need to explore alternative approaches to influenza surveillance.

We aimed to describe the feasibility, acceptability, and quality of samples collected in a home‐based self‐swabbing programme implemented in three provinces of South Africa between December 2021 and August 2022. In addition, we aimed to describe the influenza vaccine coverage and to calculate influenza vaccine effectiveness.

## 2 Methods

### 2.1 Study Setting

The study was conducted in three of the nine South African provinces. Participants were enrolled in a radius of 10 km of the field implementation sites in Hillcrest, Kwazulu‐Natal (KZN), City of Cape Town, Western Cape (WC), and Randburg, Gauteng (GauP). Individuals 18 years and older, who provided consent, self‐enrolled through a web‐based survey. We used two mechanisms for enrolling participants. By concentrating enrolment in middle and upper socioeconomic areas, we expected influenza vaccination rate to be higher than in lower socioeconomic areas where people access public health services (lower vaccine coverage).

#### 2.1.1 Cross Sectional Study (*CoughCheck*)

Individuals were recruited between 1 December 2021 and 3 September 2022 using a variety of approaches to identify individuals with respiratory symptoms including a general practitioner (GP) surveillance programme (Viral Watch [VW]), pharmacies, self‐referral to a SARS‐CoV‐2 testing centre (Epicentre), and social and printed media. Individuals were able to enrol through a web‐link; the link was provided by the GP or through pharmacists or a QR code on printed flyers in clinical facilities (GP rooms, SARS‐CoV‐2 testing Centre, pharmacies) and through local WhatsApp groups, Facebook, and other social media pages.

Once the participant opened the link and signed the consent form, they were directed to a case investigation form to confirm symptoms and complete demographic, clinical, and vaccine status data (self‐reported vaccine status). On completion of the case investigation form, the participant was able to schedule an appointment for the delivery of a sample collection kit. A driver was dispatched (Mondays to Fridays) to the participant's home with the testing kit and waited for the sample to be taken. Instructions for collecting the swab were included in the test kits, and a link to an instruction video was sent to the participant. The driver delivered the sample to the local testing laboratory on the day of collection. Participants received their test result via text message within 24 h and were contacted by an operator from the self‐referral testing centre to confirm receipt of the result and arrange referral to healthcare if needed (regardless of test result). Participants were informed of symptoms of severe illness and how to access care. Following receipt of their test result, participants received an acceptability questionnaire via email. Testing of samples was conducted at an accredited diagnostic laboratory near the study sites. Following this, samples were shipped to the reference laboratory at the National Institute for Communicable Diseases (NICD) in Johannesburg for subtyping of influenza and RSV.

#### 2.1.2 Cohort Study (*CoughWatchSA*)

Participants enrolled into (*CoughWatchSA*) a standalone digital participatory surveillance platform who reported symptoms of respiratory illness were invited to enrol into *Coughcheck*. Only participants residing in the *CoughCheck* regions who reported symptoms (eligible for enrolment into *CoughCheck)* were offered home‐based testing and sent a link to the testing study, *CoughCheck*.

### 2.2 Case Definition

We applied a broad case definition for eligibility for testing. This included any respiratory symptoms, including at least one of the following: fever (self‐reported or measured at ≥ 38°C), shortness of breath, cough, sore throat, coryza, fatigue, myalgia or chest pain, and loss of taste and/or smell.

### 2.3 Quality Control, Nurse Collected Swabs

A subsample of 100 participants had a nurse‐collected nasal swab (in addition to the self‐swab) to evaluate differences in sensitivity and specificity between nurse‐collected swabs and self‐swabbing. The nurse collected the sample at the same time as the participant‐collected sample was collected.

### 2.4 Laboratory Methods

Nasal swabs were tested for influenza, respiratory syncytial virus (RSV), and SARS‐CoV‐2 by multiplex real‐time PCR (Cepheid Xpert® Xpress CoV‐2/Flu/RSV Assay) at the KZN and WC sites and on the multiplex real‐time PCR (ThermoFisher Scientific TaqPath COVID‐19, Flu A/B, RSV assay) at the GauP testing site. Specimens testing positive on these assays were considered positive for the analysis. Specimens were then transported to NICD for confirmation using the Allplex™ SARS‐CoV‐2/FluA/FluB/RSV Assay (Seegene, Seoul, South Korea). Samples positive for influenza and RSV were further subtyped. Samples were also tested for the human RNase P gene to confirm sample quality.

### 2.5 Data Collection and Analysis

In the descriptive analysis, we reported the numbers and proportions, including the numbers of participants overall and by case status for baseline characteristics. We also describe the detection rates (proportion positive) for influenza, SARS‐CoV‐2, and RSV by week of sample collection.

We evaluated (i) the logistical feasibility and timeliness of delivering and collecting swabs and reporting results and (ii) the quality of collected samples by testing for the human RNase P gene. We estimated the sensitivity and specificity of the nurse‐collected swab compared with the participant‐collected sample. From the acceptability questionnaire, we assessed the perception of the system and participants' willingness to participate during future influenza seasons.

Vaccination was defined as reported receiving the 2022 influenza vaccination (2022 Southern Hemisphere formulation) between March 2022 and August of 2022. Vaccine effectiveness (during the influenza season of 2022) was estimated by a test‐negative design: comparing the prevalence of vaccination (any influenza vaccine received during 2022) among cases (influenza positive) and controls (influenza negative), restricting to cases and controls enrolled during the influenza season. Vaccine effectiveness was calculated as 1 − odds ratio (OR) for laboratory‐confirmed influenza in vaccinated compared with unvaccinated patients.

## 3 Results

**FIGURE 1:**
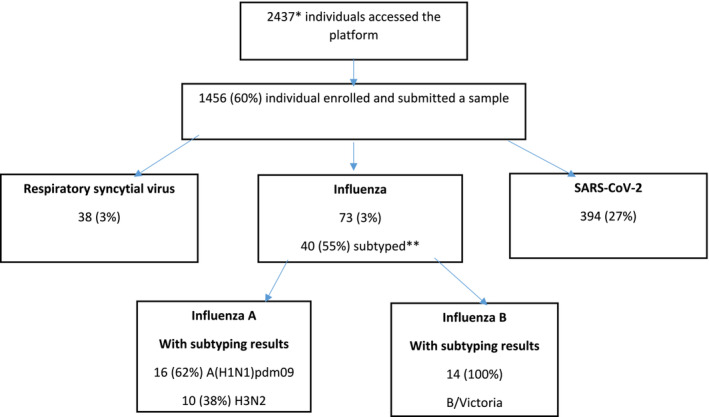
Study flow diagram, participants enrolled into the *Coughcheck* study, South Africa, December 2021 to August 2022. * includes eligible *Coughwatch* participants. **Samples were inadequate or volumes were too low after testing at the diagnostic laboratory.

Between 18 November 2021 and 3 September 2022, 2437 participants accessed the online platform and 1456 (60%) signed consent (Figure 1). All participants that signed consent returned a sample. The median age was 36 years (interquartile range [IQR] 27–51) and 64% (927/1456) were female. Older participants (≥ 65 years) accounted for 9% of enrolments (132/1456). Participant distribution across the three provinces was 32% (471/1456) in KZN, 43% (622/1456) in WC, and 25% (363/1456) in Gauteng. Sample collection kits were delivered within 24 h of enrolment for 97% of participants. For 92% (1339/1456) of participants, results were sent within 24 h of the sample being collected.

We collected and tested 1456 samples; of these, 73 (5%) tested positive for influenza, 38 (3%) tested positive for RSV, and 394 (27%) positive for SARS‐CoV‐2. Forty specimens (55%; 40/73) were available for influenza subtyping. Of these, 26/40 (65%) were influenza A (16/26, 62% A(H1N1)pdm09 and 10/26, 38% A(H3N2)), and all 14 (100%) of the influenza Bs were influenza B/Victoria (Figure 1 and Table 1).

**TABLE 1:** Participant demographic characteristics, symptoms, and viral isolation in the *CoughCheck* testing programme, South Africa 2021–2022.

	*n* (%)
**Age group (years)**	*N* = 1456
18–34	657 (45)
35–54	506 (35)
55–64	161 (11)
≥ 65	132 (9)
**Province**	*N* = 1456
KwaZulu‐Natal	471 (32)
Gauteng	363 (25)
Western Cape	622 (43)
**Enrolment link**	*N* = 1451
Clinician/pharmacy	46 (3)
Direct contact with accessible testing centre	1306 (90)
Referred from Cough Watch	99 (7)
**Vaccination status**	*N* = 1454^a^
Influenza vaccine	157 (11)
COVID‐19 vaccine	*N* = 1455
At least one COVID‐19 vaccine	1106 (76)
Janssen COVID‐19 vaccine	271/1106 (25)
Comirnaty	835/1106 (75)
**Viruses detected on PCR**	
Influenza	73/1456 (5)
Subtyping available	40/73 (55)
A(H1N1)pdm09	16/40 (40)
A(H3N2)	10/40 (25)
B/Victoria	14/40 (35)
Respiratory syncytial virus	38/1456 (3)
SARS‐CoV‐2	394/1456 (27)
Human RNAse‐P (quality of specimens)	1328/1341 (99)
**Symptoms reported**	
Duration of symptoms days (median)	2 (IQR 1–4)
ILI case definition (fever and cough)	279/1451 (19)^a^
Cough	987/1453 (68)
Fatigue	967/1452 (67)
Sore throat	902/1453 (62)
Muscle pain	773/1452 (53)
Runny nose	874/1453 (60)
Fever	367/1451 (25)
Chest pain	386/1452 (27)
Loss of appetite	427/1451 (29)
Difficulty breathing	415/1452 (29)
Diarrhoea	237/1452 (16)
Loss of taste/smell	183/1462 (13)

^a^ Data were not available for all participants, so denominator varies.

For those with data available, the median duration of symptoms prior to enrolment was 2 days (IQR 1–4). Only 19% (279/1451) of participants reported influenza‐like illness case definition symptoms of fever and cough. The most common symptoms reported were cough (987/1452, 68%), fatigue (967/1452, 67%), and sore throat (902/1453, 62%) (Table 1).

**FIGURE 2:**
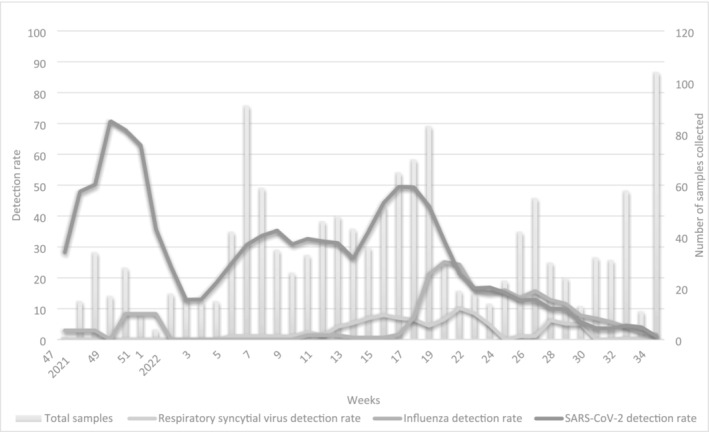
Number of specimens collected and detection rate for influenza, SARS‐CoV‐2 and respiratory syncytial virus (RSV) *Coughcheck* South Africa November 2021–September 2022.

Peak circulation of influenza was in week 22 of 2022 (42% [11/19]), RSV circulation peaked in week 24 of 2022 with 14% (2/14) and the peak of SARS‐CoV‐2 circulation was in week 52 of 2021 with 100% (10/10) testing positive for SARS‐CoV‐2 (Figure 2).

Of those enrolled, 11% (157/1454) reported receiving influenza vaccine in 2022. The highest vaccination rate was in people aged ≥ 65 years (28/132, 21%) followed by age groups 55–64 years (23/161, 14%), 35–54 years (55/506, 11%), and 18–34 years (51/655, 8%). The overall influenza VE was 26% (95% confidence interval [CI]: −73%, 69%). Characteristics of cases and controls included vaccination rate of 9% (6/70) in cases and 11% (136/1292) in controls (Table 2). Vaccination for SARS‐CoV‐2 (at least one vaccine dose) was reported by 76% (1106/1455) of participants, of whom 75% (833/1106) had received a Comirnaty and 25% (271/1106) a Janssen COVID‐19 vaccine (Table 1).

**TABLE 2:** Cases and controls included in the influenza vaccine effectiveness estimates, *Coughcheck* South Africa 2021–2022.

Variable	Cases *N* = 70	Controls *N* = 1292	Total	*p* value
	*n* (%)	*n*(%)		
			*N* = 1362	
Vaccinated for influenza	6 (9)	136 (11)	142 (10)	0.60
Unvaccinated for influenza	64 (91)	1156 (89)	1220 (90)	
Age group			*N* = 1348	
18–34	33 (47)	568 (44)	601 (33)	0.35
35–54	26 (37)	439 (34)	465 (34)	
55–64	3 (4)	139 (11)	142 (10)	
≥ 65	6 (8)	134 (10)	140 (10)	
Sex			*N* = 1362	
Male	27 (39)	468 (36)	495 (36)	0.88
Female	43 (61)	824 (64)	867 (64)	

The comparison of samples collected by a trained nurse (102 samples) at the same time as the participant resulted in a sensitivity for influenza (based on two specimens testing positive for influenza during the QC part of the study) of 98% (95% CI: 95%, 100%) and specificity of 100%. The sensitivity for SARS‐CoV‐2 was 85% (95% CI: 63%, 95%) and specificity 100% (26 positive samples on laboratory testing). RSV testing was concordant for all three of the QC specimens that tested positive. We tested 1341 (including all QC samples) specimens for RNase‐P, and 99% (1329) tested positive, verifying the integrity and quality of the specimen.

### 3.1 Enrolment and Acceptability

Only 23% (334/1454) of participants completed the acceptability questionnaire. Most participants were enrolled into the study through referral via the call‐in line at the SARS‐CoV‐2 testing centre (107/334, 32%); this was followed by word‐of‐mouth referral (80/334, 24%), social media (Facebook, WhatsApp community groups, and Instagram) (47/334, 14%) and *CoughWatchSA* participatory surveillance (30/334, 9%). Other referral mechanisms included general practitioners, pharmacists, and print media.

Procedures to enrol, give consent, and setting up delivery of sample test kits were acceptable and easy with > 95% of participants reporting these as “easy” or “very easy.” Participants reported the instructions for taking the sample were easy (> 98% reporting these procedures as “easy” or “very easy”). The majority (97%) of participants reported their willingness to participate in this kind of study in the future.

## 4 Discussion

In our study of home‐based collection of nasal swabs, we were able to identify three commonly circulating respiratory viruses and estimate a VE for the annual influenza vaccine. In addition, the study was acceptable to study participants and more than 92% received their test result within 24 h. A QC substudy showed good concordance with self‐collected samples. Although home‐based testing with nasal swabs is not a new concept, it was important to confirm this in South Africa, a low‐middle income setting. This community‐based surveillance approach may provide an important platform to access vaccinated populations and individuals with non–medically attended illness.

Home‐based swabbing programmes have been effective in developed countries. In the United States, a study of home‐based testing for influenza (including the starting of anti‐viral medication) showed good sensitivity and specificity of rapid tests and allowed early commencement of antiviral medication [5]. In Hong Kong, a study validating self‐swabbing confirmed it was possible to identify viruses from self‐swabbing samples including quantitative testing of viral load [6].

The identification of circulating viruses mapped the seasonal peak of influenza in week 22, compared with week 23 in our other surveillance programmes, including a general practitioner–based surveillance programme, Viral Watch [7]. Similarly, this study identified an increase in RSV circulation between week 13 and 24, compared with week 11 and 25 in other surveillance programmes [7]. The RSV season in South Africa is usually described based on children < 5 years admitted to hospital; therefore, the lower detection rate in this study may be explained by the fact that this study enrolled adults only in an outpatient setting. Home‐based testing also marked the Omicron variant wave in December 2021 as in our other surveillance programmes [8].

The VE point estimates in this study were lower than those of the Viral Watch programme, although CIs were very wide due to low numbers. There are a number of possible reasons for this: We relied on self‐reported vaccine status and collected only a month of vaccination (not day); this did not allow us to exclude individuals who were vaccinated within 14 days of their symptoms (as is done in the Viral Watch analysis). This may explain the difference in the VE point estimates as compared with the estimates in the Viral Watch programme (65%, 95% CI: 30%, 82%), adjusted for age and season [9]. The precision around the point estimate for this study is low, likely due to low numbers of vaccinated people. Self‐reporting of vaccine status may also introduce recall bias, either by not reporting vaccination or incorrectly reporting the year of vaccination. These estimates may improve if similar data points were collected in both the *Coughcheck* and Viral Watch programmes allowing pooling of data.

Several international studies report excellent concordance between self‐swabbing and practitioner‐collected samples. Ip et al. report good concordance for influenza viral load detected on self‐swabbing compared with nurse‐collected samples for all subtypes of influenza, and through day 0 to day 12 of infection [6]. We confirmed this in our setting with very good concordance between self‐administered nasal swabs and nurse‐administered swabs. In a similar study, conducted in Germany, the authors reported that the study was acceptable in participants of all ages and found high detection rates of pathogens and confirmed the presence of human DNA on the swab in 71% of symptomatic patients [10]. In our study, we found even higher RNase P positive rates of 98%.

Our study had several limitations. Although we were able to streamline the enrolment processes by including easy accessible web‐links and QR codes, delivery and collection of samples was labour and cost intensive. This may affect the sustainability of this type of surveillance programme in our setting. This also limited enrolment to a 10‐km radius of the testing centre. We were not able to subtype all of the influenza positive specimens due to a proportion of specimens not having adequate volume or quality after the diagnostic laboratory completed testing; in the future, we plan to test at NICD only. The proportion of influenza subtypes in this study was however similar to our other surveillance programmes for the study period [7]. Self‐reporting of influenza vaccination status may have biased estimates of influenza VE and included incomplete dates of vaccination, which may have underestimated the VE. We enrolled relatively few older people (only 9% of participants were ≥ 65 years), this may have introduced bias in to our VE estimates. It is possible that the digital nature of the enrolment and the home visits may have deterred older people. We conducted this study during the SARS‐CoV‐2 pandemic and the interest in testing was high. Different strategies may be needed to continue this surveillance during the annual influenza season, when demand for testing may not be as high.

## 5 Conclusions

A home‐based self‐swabbing surveillance programme may add an additional element to surveillance programmes, specifically accessing a vaccinated population. These data would add to a body of data to inform vaccine strain selection and support the strengthening of influenza vaccine programmes.

## Author Contributions

All authors have contributed to and reviewed the manuscript. Jocelyn Moyes: study design, protocol preparation, data analysis, preparation of the manuscript. Mvuyo M. Makhazi: data management and analysis. Sibongile Walaza: study design, protocol preparation. Phiwokuhle Ntombela: data management. Fahima Moosa: laboratory testing, data management. Anne von Gottberg: study design, oversight of laboratory process. Nicole Wolter: laboratory scientific input, study design. Mignon du Plessis: laboratory scientific input. Gillian Hunt: diagnostic laboratory oversight, scientific input. Cherie Cawood: field work implementation, site data management. Erica Dueger: study design, preparation. Cheryl Cohen: study design, protocol preparation, preparation of the manuscript.

## Ethics Statement

This project received approval from the Human Research Ethics Committee of the University of the Witwatersrand (210414). All participants provided electronic consent prior to starting the project procedures.

## Conflicts of Interest

Jocelyn Moyes has received funding from Sanofi and the Bill and Melinda Gates Foundation. Nicole Wolter has received grant funding from the Bill and Melinda Gates Foundation and Sanofi. Cheryl Cohen has received grant funding from the US Centers for Disease Control and Prevention, Wellcome, Taskforce for Global Health, Bill and Melinda Gates Foundation and Sanofi. Sibongile Walaza has received grant funding from US Centers for Disease Control and Prevention, Taskforce for Global Health and Bill and Melinda Gates Foundation. Mignon du Plessis received a grant from the Bill and Melinda Gates Foundation. E Dueger is a Sanofi employee and may hold stock and/or stock options.

### Peer Review

The peer review history for this article is available at https://www.webofscience.com/api/gateway/wos/peer‐review/10.1111/irv.70034.

## Data Availability

The data that support the findings of this study are available from the corresponding author upon reasonable request.
